# Predictive value of uric acid and lactate dehydrogenase for maternal morbidity in preeclampsia: a retrospective case-control study

**DOI:** 10.3389/fmed.2026.1750426

**Published:** 2026-03-26

**Authors:** Çağri Kutlugün Emral, Emre Sinan Güngör, Yusuf Ziya Kizildemir

**Affiliations:** 1 Department of Obstetrics and Gynecology, Sanliurfa Training and Research Hospital, Sanliurfa, Türkiye; 2 Department of Obstetrics and Gynecology, Istanbul Education and Research Hospital, Istanbul, Türkiye

**Keywords:** acute kidney injury, lactate dehydrogenase, maternal morbidity, preeclampsia, uric acid

## Abstract

**Objective:**

Preeclampsia is a major cause of global maternal morbidity and mortality. This study aims to determine whether readily available and inexpensive biomarkers, serum uric acid (UA), and lactate dehydrogenase (LDH), show a significant association with severe maternal morbidity in established preeclampsia.

**Materials and methods:**

In this retrospective Case-Control study (2017-2022), 200 singleton pregnancies with ACOG-defined preeclampsia and 200 age and gestational age-comparable healthy controls delivered at a tertiary center were analyzed. UA and LDH were serially measured (minimum, average, maximum). Maternal morbidities (acute kidney injury, pulmonary oedema, neurological seizures, vision loss) were adjudicated using KDIGO, Berlin and ACOG criteria.

**Exclusions:**

HELLP syndrome, chronic hypertension, diabetes, renal/liver/cardiac disease, systemic infection. Multivariable logistic regression adjusted for age, BMI, parity, and mean systolic BP.

**Results:**

Mean UA (5.8 ± 1.3 vs. 4.4 ± 1.1 mg/dL) and LDH (410.7 ± 194.0 vs. 267.2 ± 92.0 IU/L) were higher in preeclampsia (*p* < 0.001). BMI was also higher in the preeclampsia group (29.5 ± 4.2 vs. 26.1 ± 3.8 kg/m^2^, *p* < 0.001). UA independently predicted acute kidney injury (aOR 3.21, 95 % CI 2.50–4.12) at 6.3 mg/dL (AUC 0.924, 97.8 % NPV). UA ≥ 8.0 mg/dL was 100 % sensitive for seizures; UA ≥ 6.1 mg/dL for vision loss (90.9 % sensitivity). Additionally, an average LDH level ≥ 516.4 IU/L showed high utility for ruling out pulmonary edema (99.4% NPV). LDH did not retain independent predictive significance for most outcomes in multivariable modeling. No preeclampsia-related deaths occurred.

**Conclusion:**

In a Middle Eastern cohort, UA is an inexpensive, widely available marker that is significantly associated with severe maternal morbidity in established preeclampsia, whereas LDH, while reflecting disease severity, did not add independent predictive value for most outcomes after adjustment. Prospective multicentre validation is required.

## Introduction

Preeclampsia is one of the most significant causes of mortality and morbidity during pregnancy. In this study, preeclampsia was defined according to the revised criteria of the International Society for the Study of Hypertension in Pregnancy (ISSHP), which includes new-onset hypertension after 20 weeks of gestation accompanied by proteinuria or other maternal organ dysfunction, such as renal insufficiency, liver involvement, or neurological complications. On a global scale, preeclampsia affects 4–6% of all pregnancies (1). Moreover, the burden of this disease is disproportionately high in low- and middle-income countries, where it is associated with a significantly higher risk of maternal mortality compared to high-income nations (2). The prevalence of preeclampsia varies based on factors such as age, race, nulliparity, pre-existing chronic diseases, obesity, and lifestyle (3). As the number of risk factors increases, there is a corresponding increase in the incidence of preeclampsia (4). The maternal and fetal outcomes of increasing preeclampsia cases can be severe, and the burden on healthcare systems in terms of both workforce and financial costs cannot be overlooked. Preeclampsia is widely recognized as an endothelial cell disorder, characterized by systemic endothelial injury that impairs vasorelaxing and anticoagulant pathways. Placental factors released into the circulation due to poorly perfused tissue are believed to initiate this dysfunctional cascade of vasoconstriction and coagulation, which manifests as the clinical syndrome of preeclampsia.

Recent studies have focused on preventive healthcare policies, especially in obstetrics and gynecology, where screening and diagnostic tests for prevention have gained importance. In obstetrics, predicting the severity and outcomes of preeclampsia in advance is crucial. However, no screening test or imaging technique has yet been directly associated with the severity or outcomes of the disease. Commonly used methods, such as monitoring protein levels in urine and measuring blood pressure during routine check-ups, are also insufficient for predicting outcomes (5). Therefore, research into effective screening methods continues.

The Fetal Medicine Foundation has developed a risk prediction tool that combines average blood pressure, uterine artery resistance, and serum PIGF (placental growth factor) and PAPP-A (pregnancy-associated plasma protein-A) levels to predict the risk of preeclampsia (6). However, these tests only indicate the risk of developing preeclampsia and do not provide information about the potential mortality and morbidity outcomes of the disease.

In the context of preeclampsia, identifying accessible and reliable biomarkers for risk stratification is crucial (7). Lactate dehydrogenase (LDH) stands out as an easily accessible and cost-effective biomarker, widely recognized for its indication of endothelial damage, hemolysis, and cellular destruction stemming from the systemic inflammatory response characteristic of preeclampsia. Similarly, uric acid (UA) is a well-established biomarker that reflects kidney damage often seen in preeclampsia and can exert toxic effects above certain concentrations. Given their direct involvement in the pathophysiological processes of preeclampsia and their ready availability in most healthcare settings, UA, and LDH were selected for this study. Previous research has indeed suggested that levels of these biomarkers, alongside others like ALT, may be significant in predicting the prognosis of preeclampsia. Therefore, this study specifically aimed to investigate and correlate the levels of UA and LDH with maternal mortality and morbidity caused by preeclampsia, with the ultimate goal of identifying more accessible and meaningful diagnostic tools for predicting disease outcomes (8). This highlights a critical gap: the need for prognostic markers that are not only reliable but also universally available and affordable. Therefore, while newer biomarkers like placental growth factor (PlGF) are valuable, this study focused on UA and LDH due to their cost-effectiveness and widespread availability, making them highly applicable in diverse clinical settings, including those with limited resources (9). While UA and LDH are established markers, this study provides novel clinical utility by deriving specific diagnostic cut-offs with high negative predictive values (NPV) tailored for a Middle Eastern cohort, offering a practical risk-stratification tool for resource-limited settings where advanced biomarkers are unavailable.

### Primary objective

To evaluate the independent association between UA and LDH levels and severe maternal morbidities in women with established preeclampsia. Secondary objectives: to derive clinically useful cut-offs for these biomarkers and to assess their negative-predictive utility for resource-limited settings.

Based on this rationale, we hypothesized that elevated serum levels of UA and LDH in patients with preeclampsia would be significantly correlated with, and predictive of, specific severe maternal morbidities, including acute kidney injury, pulmonary edema, neurological seizures, and vision loss.

## Materials and methods

### Study design and ethical approval

This study was designed as a retrospective case-control analysis within a larger cohort framework to evaluate the relationship between biomarkers and maternal morbidity. The study was conducted in accordance with the ethical standards of the institutional and national research committee and with the 1964 Helsinki Declaration and its later amendments. The study protocol was approved by the Ethics Committee of the Istanbul Health Practice and Research Center, University of Health Sciences on 28.04.2023 (Academic Board Decision No. 237737-51/23). Informed consent was not obtained due to the retrospective nature of the study involving the use of anonymized patient data.

### Participants

This retrospective cohort study included 200 pregnant women diagnosed with preeclampsia who were admitted to the Obstetrics and Gynecology Clinic of the Istanbul Education and Research Hospital between 2017 and 2022, a period during which comprehensive electronic health records were consistently available. For the control group, 200 healthy pregnant women were selected from consecutive deliveries immediately following each preeclamptic patient (defined as delivering within the same 24 h period) to minimize temporal variations in hospital protocols. However, we explicitly acknowledge that this consecutive selection method carries a risk of selection bias and residual confounding, as it does not provide the robust control offered by a matched design or propensity score matching. Consequently, the observed differences between the groups should be interpreted with caution.

#### Inclusion criteria

Age between 18 and 45 years; A diagnosis of preeclampsia for the patient group, no diagnosis of preeclampsia for the control group; Delivery at our hospital; and singleton pregnancies with complete UA/LDH data within 48 h of delivery.

#### Exclusion criteria

HELLP syndrome; chronic hypertension; chronic kidney disease; chronic endocrine diseases (e.g., diabetes mellitus); chronic liver disease; autoimmune diseases (e.g., systemic lupus erythematosus); gestational diabetes mellitus or cholestasis of pregnancy; systemic infection; patients who refused treatment or were discharged against medical advice (as their outcomes could not be fully ascertained); and gestational age < 24 weeks. To isolate the impact of preeclampsia-related hemolysis on LDH levels, we reviewed peripheral blood smears and indirect bilirubin levels where available. Patients with primary hematological disorders (e.g., sickle cell anemia, autoimmune hemolytic anemia) were excluded to prevent confounding of LDH results.

### Data collection and variables

Patient data, such as age, geographic distribution, gravidity, parity, delivery method, all LDH and UA levels (minimum, maximum, and average values), the average of all systolic and diastolic blood pressure measurements during hospitalization, and pre- and post-delivery hemoglobin levels were retrieved retrospectively from the hospital's PROBEL Medical Information and Management System. For laboratory measurements, the Cobas 6000 analyzer (c501 module, Roche Diagnostics, Mannheim, Germany) was used. In addition to UA, other renal function markers including serum creatinine and Blood Urea Nitrogen (BUN) were routinely monitored for all patients as part of the institutional protocol for preeclampsia management. The reference ranges for uric acid (2.5–5.7 mg/dL) and LDH (135–214 IU/L) were based on our institution's validated laboratory standards. All blood samples were collected as part of routine clinical care without standardized fasting requirements. Given the retrospective nature, fasting status was not consistently documented. While this may introduce measurement variability, the same collection protocols applied to both preeclampsia and control groups, minimizing systematic bias. To clarify the timing, while inclusion required at least one complete dataset within 48 h of delivery, our analysis utilized all available measurements during the patient's hospitalization (e.g., at admission, pre-delivery, and post-delivery) to calculate the minimum, average, and maximum values, reflecting the total burden of the disease. Factors that could potentially affect UA and LDH levels (e.g., pre-delivery blood transfusions, massive bleeding, dehydration, active infections) were excluded.

### Definitions of maternal morbidity

Preeclampsia was defined according to the criteria of the American College of Obstetricians and Gynecologists (ACOG). All specific maternal morbidities examined in this study were diagnosed using standardized clinical and laboratory criteria, as detailed in Table 1.

**Table 1:** Definitions and diagnostic criteria for maternal morbidities.

**Morbidity**	**Diagnostic criteria**
Acute kidney injury	Defined as a ≥0.3 mg/dL increase in serum creatinine within 48 h or a serum creatinine level ≥1.5 times the baseline value, according to the KDIGO criteria.
Pulmonary edema	Diagnosed based on clinical signs (e.g., dyspnea, hypoxia) combined with radiographic evidence of bilateral infiltrates on a chest X-ray.
Pneumonia	Required the presence of a new pulmonary infiltrate on imaging plus at least two clinical signs, such as fever (>38 °C), leukocytosis (>12,000/μL), or positive sputum culture.
Thromboembolism	Confirmed by Doppler ultrasound for deep vein thrombosis (DVT) or CT pulmonary angiography for pulmonary embolism (PE).
Wound Infection	Diagnosed by the presence of purulent discharge, erythema, or tenderness at the surgical site, accompanied by fever (>38 °C) or a positive wound culture.
Neurological seizures (Eclampsia)	Defined as new-onset tonic-clonic seizures in a preeclamptic patient. Diagnosis was made clinically by the attending obstetrician and confirmed via neurology consultation, which excluded other causes (e.g., epilepsy, stroke). Specific neuro-imaging or EEG was not applied as a standard diagnostic criterion for all cases, aligning with the ACOG definition of eclampsia.
Vision loss	Documented based on the patient's report of sudden bilateral blurred vision, confirmed by a retinal examination showing findings like vasospasm or papilledema.
ICU admission	Defined as admission to the ICU for ≥24 h due to preeclampsia-related complications (e.g., refractory hypertension, organ failure).

### Statistical analysis

Statistical analyses were performed using SPSS 29.0 software. The normal distribution of continuous variables was evaluated with the Kolmogorov-Smirnov test. Descriptive statistics were expressed as numbers (*n*) and percentages (%) for categorical variables, and as mean standard deviation for continuous variables. As continuous variables were not normally distributed, the non-parametric Mann-Whitney U test was used for analytical comparisons between groups. The ROC curve was employed to determine the predictive cutoff values of serum LDH and serum uric acid levels for specific complications. Youden's index was used to determine the cutoff points. To assess the independent predictive value of biomarkers while controlling for confounders (maternal age, BMI, parity, and mean systolic blood pressure), a binary logistic regression analysis was performed for significant morbidities. The analysis included only patients with complete data for the primary variables of interest. A *p*-value of less than 0.05 was considered statistically significant. Although formal *a-priori* power calculation was not feasible in this retrospective design, a *post-hoc* power analysis indicated 92% power (α = 0.05) to detect an aOR ≥ 2.5 for AKI with UA ≥ 6.3 mg/dL, suggesting adequate sample size for this primary outcome. Internal validity of cut-offs was explored by bootstrapping (1000 iterations). Multicollinearity in the regression models was assessed using the Variance Inflation Factor (VIF), with a VIF > 5 considered problematic. Given the low frequency of certain clinical events, specifically eclamptic seizures and pulmonary edema, the multivariable models and derived cut-off values for these outcomes are categorized as exploratory. These results serve as hypothesis-generating data rather than definitive clinical thresholds.

## Results

**Figure 1:**
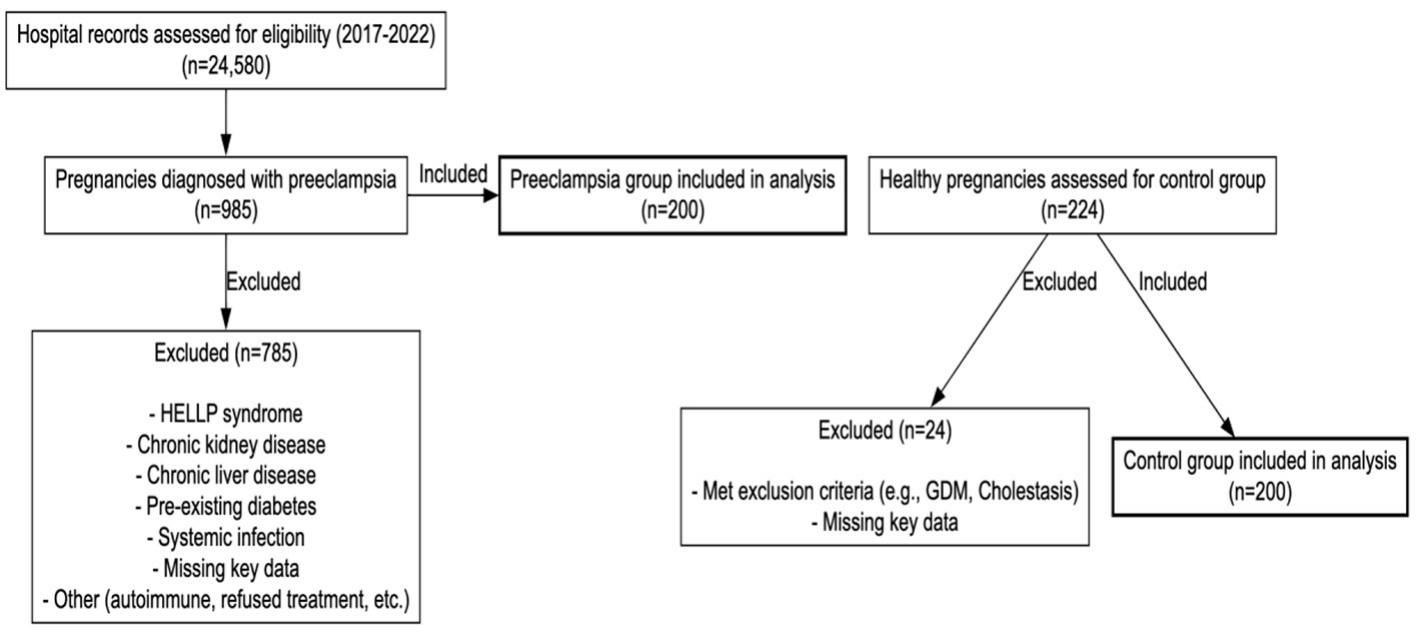
STROBE flow diagram of patient selection.

The patient selection process according to the STROBE guidelines is detailed in Figure 1. Initially, 24,580 hospital records were assessed for eligibility between 2017 and 2022. Among these, 985 pregnancies were diagnosed with preeclampsia. Following the application of strict exclusion criteria, 785 patients were excluded primarily due to HELLP syndrome, chronic hypertension, pre-existing diabetes, or missing key laboratory data resulting in a final preeclampsia cohort of 200 cases. For the control group, 224 healthy pregnancies were assessed, and 24 were excluded due to missing data or meeting exclusion criteria, leaving 200 healthy controls for analysis. This rigorous selection process ensures a high degree of cohort homogeneity for evaluating UA and LDH utility. In the study groups, the average age of the 200 patients in the preeclampsia group was 30.6 ± 6.5, while the average age in the control group was 29.3 ± 6.0. Age did not show a statistically significant difference between the two groups (*p* = 0.077). While age and parity were comparable, the preeclampsia group had a significantly higher mean BMI (29.5 ± 4.2 vs. 26.1 ± 3.8 kg/m^2^, *p* < 0.001) (Table 2). To account for this, BMI was included as a covariate in all multivariable logistic regression models to ensure the independent predictive value of UA and LDH.

**Table 2:** Demographic and clinical characteristics of the study groups.

**Variable**	**Case group (*n* = 200)**	**Control group (*n* = 200)**	*p*-value^*^
Age (years)	30.6 ± 6.5 (18.2–43.7)	29.3 ± 6.0 (18.5–43.3)	0.077
Gravida	2.3 ± 1.5 (1.0–8.0)	2.6 ± 1.5 (1.0–8.0)	0.006
Parity	2.0 ± 1.3 (0.0–6.0)	2.3 ± 1.2 (1.0–6.0)	< 0.001
Body mass index (kg/m^2^)	29.5 ± 4.2	26.1 ± 3.8	< 0.001
Mean systolic BP (mmHg)	148.0 ± 7.8 (132.0–172.0)	119.2 ± 12.4 (97.0–139.0)	< 0.001
Mean diastolic BP (mmHg)	87.1 ± 6.7 (72.0–108.0)	74.3 ± 6.7 (56.0–91.0)	< 0.001

Data are presented as mean ± standard deviation (minimum–maximum). *p*-values were calculated using the Mann-Whitney U Test. BMI, body-mass index; q-values after Benjamini–Hochberg correction. ^*^p < 0.05, indicates statistically significant difference between groups.

The geographic distribution of the patients in the preeclampsia group showed that 158 (79%) were of Middle Eastern origin, 30 (15%) were of African origin, 11 (5.5%) were of Asian origin, and 1 (0.5%) was of European origin. In the control group, 173 (86.5%) were of Middle Eastern origin, 17 (8.5%) were of African origin, and 10 (5%) were of Asian origin. Regarding the mode of delivery in the preeclampsia group, 31 patients (15.5%) delivered vaginally, and 169 patients (84.5%) underwent cesarean section. In contrast, 80 patients in the control group (40%) delivered vaginally, while a total of 120 patients (60%) delivered by cesarean section. The study also compared the use of erythrocyte suspension and fresh frozen plasma between the preeclampsia and control groups.

In the preeclampsia group, 36 out of 200 patients (18%) experienced acute kidney injury, whereas only two patients (1%) in the control group had acute kidney injury. Pulmonary edema was observed in seven patients (3.5%) in the preeclampsia group, but none in the control group. In the preeclampsia group, three patients (1.5%) were diagnosed with pneumonia, while none were diagnosed with pneumonia in the control group. In the control group, only one patient (0.5%) developed thromboembolism, whereas four patients (2%) in the preeclampsia group experienced thromboembolism. Wound infections occurred in four patients (2%) in the preeclampsia group and two patients (1%) in the control group. Eclamptic seizures were detected in four patients (2%) in the preeclampsia group. In the control group, two patients (1%) experienced seizures unrelated to hypertension (one patient had pre-existing epilepsy and was excluded from this specific outcome analysis, and another was diagnosed with sagittal sinus thrombosis). In the preeclampsia group, 22 patients (11%) experienced vision loss, but no vision loss was reported in the control group. In the preeclampsia group, 60 patients (30%) were followed in the ICU. Of these, 56 (93.3%) were admitted due to resistant hypertension. In the control group, only 10 patients (5%) were monitored in the ICU for various reasons.

### Treatment details

In the preeclampsia group, 156 patients (78%) received magnesium sulfate for seizure prophylaxis. Antihypertensive medications were administered to 148 patients (74%): labetalol (*n* = 89, 60.1%), extended-release nifedipine (*n* = 35, 23.6%), and hydralazine (*n* = 24, 16.2%). Specific antihypertensive regimens and their relationship to biomarker levels could not be systematically analyzed due to the retrospective design.

### Comparison of preeclampsia and control groups

The preeclampsia and control groups were compared in terms of age, gravidity, parity, LDH levels (minimum, maximum, and average), UA levels (minimum, maximum, and average), systolic and diastolic blood pressure. The average age was 30.6± 6.5 years in the preeclampsia group and 29.3± 6.0 years in the control group. There was no statistical difference between the ages of the groups (*p* > 0.05). When comparing gravidity and parity, the average gravidity was 2.3 ± 1.5 and the average parity was 2.0 ± 1.3 in the preeclampsia group, while in the control group, the gravidity was 2.6 ± 1.5 and the parity was 2.3 ± 1.2. These differences were statistically significant (*p* < 0.05). There were also significant differences between the groups in terms of UA, LDH, and blood pressure values. In the preeclampsia group, the minimum LDH level was 279.2± 93.1, the average LDH level was 410.7± 194.0, and the maximum LDH level was 555.2± 303.3. In the control group, the minimum LDH level was 228.3± 73.6, the average LDH level was 267.2± 92.0, and the maximum LDH level was 314.8± 137.3. There were statistically significant differences between the minimum, average, and maximum LDH levels in the preeclampsia and control groups (*p* < 0.05). Similarly, the minimum UA level in the preeclampsia group was 5.1± 1.2, the average UA level was 5.8± 1.3, and the maximum UA level was 6.3± 1.5. In the control group, the minimum UA level was 4.1± 1.1, the average UA level was 4.4± 1.1, and the maximum UA level was 4.7± 1.2. These differences were also statistically significant (*p* < 0.05), suggesting that monitoring UA and LDH levels could be meaningful in preeclampsia cases (Table 3).

**Table 3:** Comparison of laboratory findings between the study groups.

**Variable**	**Case group (*n* = 200)**	**Control group (*n* = 200)**	*p*-value^*^
LDH (mean, IU/L)	410.7 ± 194.0 (202.3–1,891.0)	267.2 ± 92.0 (137.5–1,072.8)	< 0.001
Uric acid (mean, mg/dL)	5.8 ± 1.3 (2.3–9.6)	4.4 ± 1.1 (1.8–7.8)	< 0.001
Hemoglobin (antepartum, g/dL)	11.5 ± 1.6 (7.7–14.6)	11.5 ± 1.3 (7.5–14.6)	0.335
Hemoglobin (postpartum, g/dL)	9.8 ± 1.5 (6.6–13.0)	9.8 ± 1.4 (6.2–13.2)	0.514

Data are presented as mean ± standard deviation (minimum–maximum). *p*-values were calculated using the Mann-Whitney U Test. ^*^p < 0.05, indicates statistically significant difference between groups.

When comparing blood pressure values between the two groups, there were significant differences in both maximum and average systolic and diastolic blood pressure values (*p* < 0.05). This result was expected given the pathogenesis and pathophysiology of the disease. However, no significant difference was found between the two groups in pre- and post-delivery hemoglobin levels.

### ROC analysis and case group evaluation

**Figure 2:**
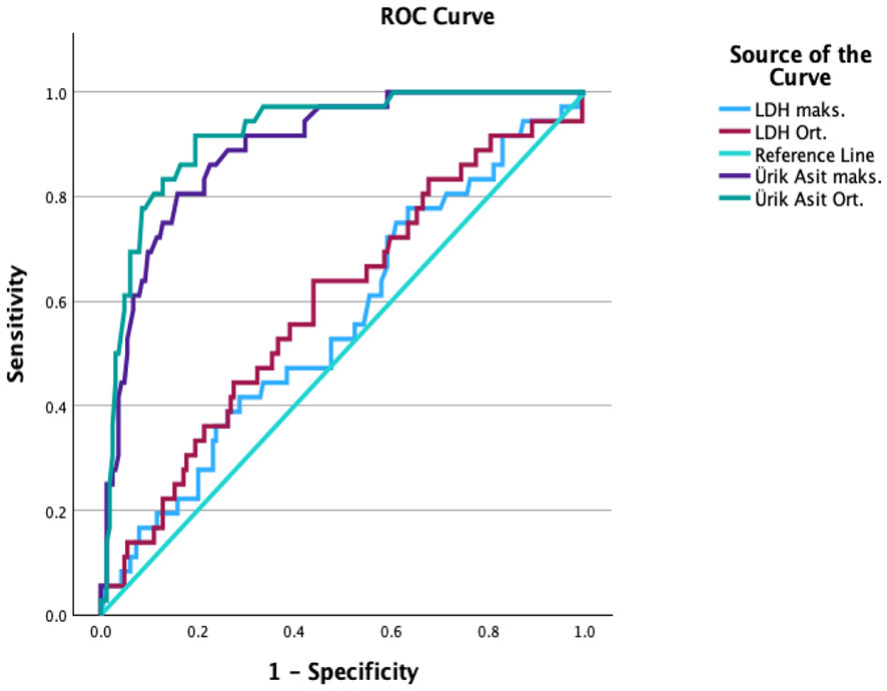
ROC Analysis of acute kidney injury/failure.

**Figure 3:**
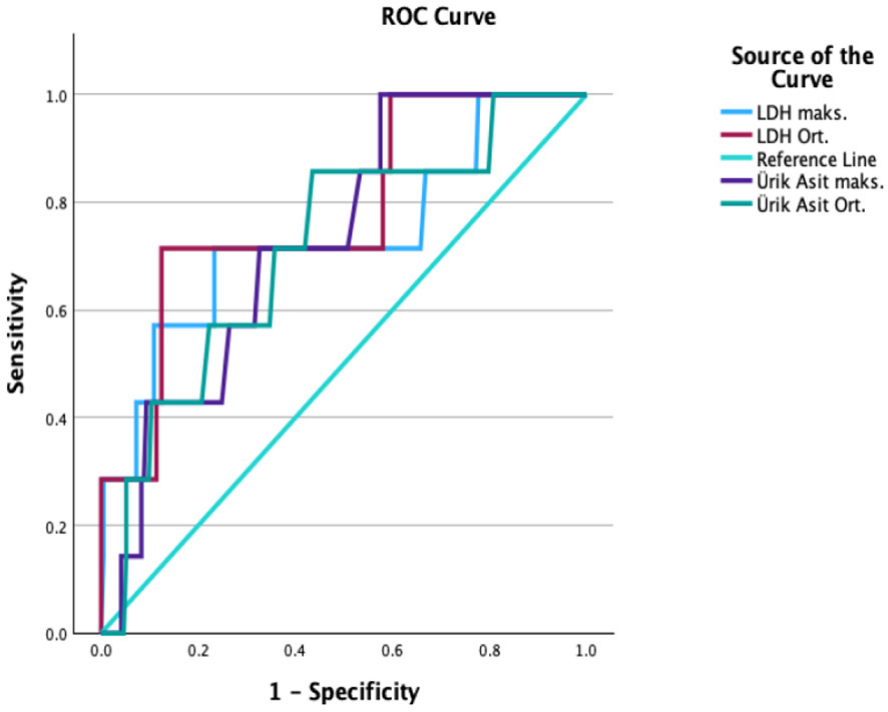
ROC Analysis for pulmonary edema.

**Figure 4:**
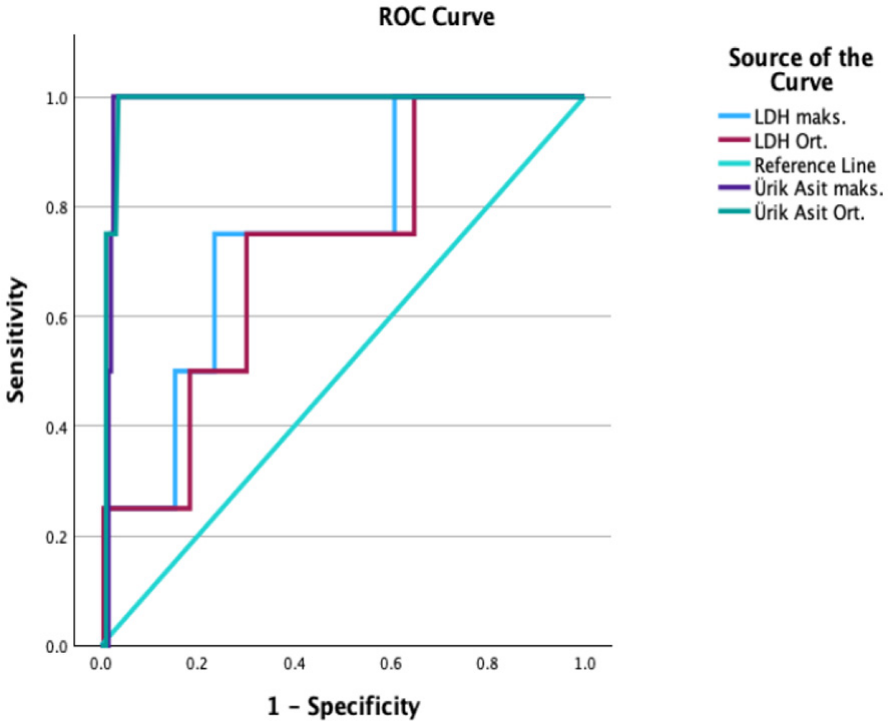
ROC analysis for neurological seizures.

**Figure 5:**
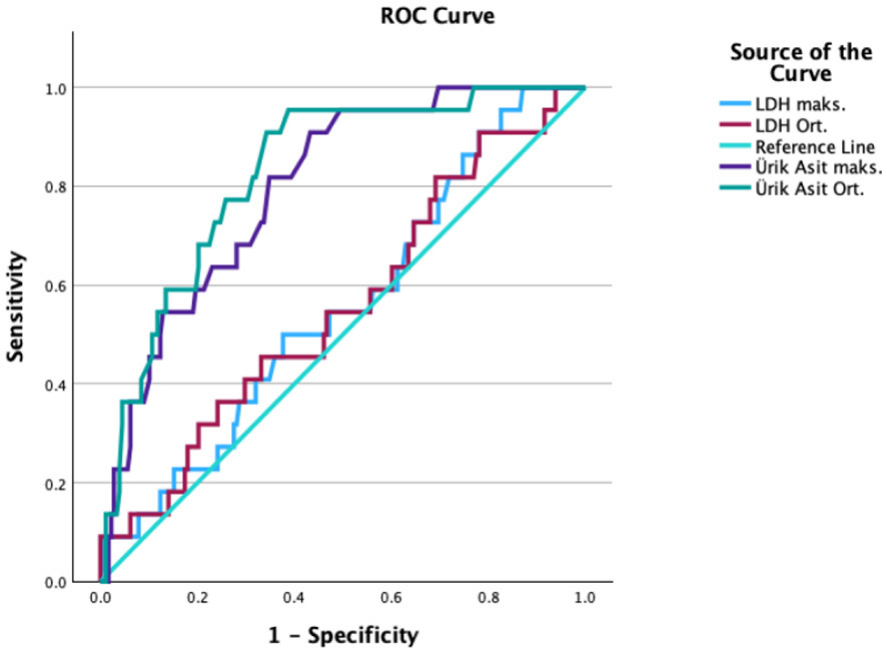
ROC analysis for vision loss.

In our study, the morbidities that arose were treated in a timely and effective manner without leaving permanent sequelae. During the course of the study, only one mortality occurred in the Obstetrics and Gynecology Clinic of Istanbul Education and Research Hospital, which was determined to be caused by an amniotic fluid embolism after further investigation. There were no preeclampsia-related mortalities, which is a positive outcome from our perspective. This positive result can be attributed to the fact that our hospital is a tertiary healthcare institution, and our team responded to the condition promptly and effectively. After comparing the case and control groups, a ROC analysis (Receiver Operating Characteristic Curve) was performed to evaluate the relationship between LDH and UA levels and morbidity in the case group. The analysis for pneumonia, thromboembolism, and wound site infection did not yield statistically significant results in relation to LDH and UA levels (all *p* > 0.05), as these morbidities showed weak or no correlation with the biomarkers and the AUC values were not predictive. However, statistically significant results were obtained when comparing UA and LDH levels with acute kidney injury, pulmonary edema, neurological seizures, and vision loss. The detailed results of the ROC analysis, including diagnostic performance metrics and correlation coefficients for these significant findings, are presented in Table 4 and Figures 2–5.

**Table 4:** Diagnostic performance and correlation of biomarkers in predicting preeclampsia complications.

**Morbidity**	**Biomarker**	**Cutoff value**	**AUC (95% CI)**	**Sensitivity (%)**	**Specificity (%)**	**PPV (%)**	**NPV (%)**	**Correlation (ρ)**	***p*-value**
Acute kidney injury	Average uric acid	6.3 mg/dL	0.924 (0.895–0.953)	91.7	81.5	52.3	97.8	0.65	< 0.001
Acute kidney injury	Maximum uric acid	7.3 mg/dL	0.895 (0.859–0.931)	80.6	84.1	52.7	95.2	0.61	< 0.001
Pulmonary edema	Average LDH	516.4 IU/L	0.840 (0.761–0.919)	71.4	87.6	15.6	99.4	0.38	< 0.05
Pulmonary edema	Maximum uric acid	5.8 mg/dL	0.715 (0.612–0.818)	100	42.5	6.0	100	0.45	< 0.05
Neurological seizures	Average uric acid	8.0 mg/dL	0.985 (0.960–1.000)	100	96.4	35.7	100	0.70	< 0.001
Neurological seizures	Maximum uric acid	8.8 mg/dL	0.990 (0.971–1.000)	100	97.4	44.4	100	0.72	< 0.001
Vision loss	Average uric acid	6.1 mg/dL	0.855 (0.798–0.912)	90.9	65.7	25.6	98.3	0.51	< 0.001
Vision loss	Maximum uric acid	6.4 mg/dL	0.810 (0.733–0.887)	90.0	56.7	20.9	98.0	0.48	< 0.001

PPV, positive predictive value; NPV, negative predictive value. Spearman's rho (ρ) was used as the correlation coefficient. AUC, area under the curve; CI, confidence interval.

For acute kidney injury/failure, the diagnostic performance of LDH and UA levels was analyzed as summarized in Table 3. While LDH levels did not show significant predictive value (*p* > 0.05), UA levels were highly significant. For average UA levels, a cutoff of 6.3 mg/dL demonstrated strong predictive ability with an AUC of 0.924, showing 91.7% sensitivity, 81.5% specificity, a 52.3% positive predictive value (PPV), and a 97.8% negative predictive value (NPV) (*p* < 0.001) (Figure 2). Moreover, a strong, positive correlation was found between average UA levels and the development of acute kidney injury (ρ = 0.65, *p* < 0.001).

For pulmonary edema, both LDH and UA levels showed significant predictive value (Table 3). An average LDH level cutoff of 516.4 IU/L had a 99.4% NPV, indicating its utility in ruling out pulmonary edema. For maximum UA levels, a cutoff of 5.8 mg/dL yielded 100% sensitivity and a 100% NPV, though the PPV was low (6.0%) due to the condition's low prevalence (Figure 3). A moderate positive correlation was observed between maximum UA levels and pulmonary edema (ρ = 0.45, *p* < 0.05).

For predicting neurological seizures, UA levels were found to be a very strong predictor (Table 3). An average UA level of 8.0 mg/dL and a maximum level of 8.8 mg/dL both showed 100% sensitivity and 100% NPV. The PPV for a maximum UA level was 44.4% (Figure 4). A very strong positive correlation was identified between maximum UA levels and neurological seizures (ρ = 0.72, *p* < 0.001).

For vision loss, UA levels were again a significant predictor (Table 3). An average UA level cutoff of 6.1 mg/dL showed 90.9% sensitivity and a high NPV of 98.3%. This indicates that patients with UA levels below this threshold are highly unlikely to experience vision loss (Figure 5). A strong positive correlation was found between average UA levels and vision loss (ρ = 0.51, *p* < 0.001).

### Multivariate logistic regression analysis

All regression models demonstrated stability, with no significant multicollinearity detected (all VIF values < 2.0). To assess whether UA and LDH levels were independent predictors of morbidity, a binary logistic regression analysis was performed, adjusting for potential confounders including maternal age, BMI, parity, and mean systolic blood pressure. The results are presented in Table 5.

**Table 5:** Multivariate logistic regression analysis for predicting maternal morbidity (Adjusted for age, BMI, parity, and mean systolic BP).

**Morbidity**	**Biomarker (per 1 unit increase)**	**Adjusted Odds Ratio (aOR)**	**95% Confidence interval (CI)**	***p*-value**
Acute kidney injury	Average uric acid (mg/dL)	3.21	2.50–4.12	< 0.001
	Average LDH (IU/L)	1.001	0.998–1.004	0.450
Pulmonary edema	Average uric acid (mg/dL)	1.70	1.12–2.58	0.012
	Average LDH (IU/L)	1.003	1.001–1.006	0.009
Neurological seizures	Average uric acid (mg/dL)	4.10	2.95–5.70	< 0.001
	Average LDH (IU/L)	1.002	0.997–1.007	0.380
Vision loss	Average uric acid (mg/dL)	2.25	1.62–3.13	< 0.001
	Average LDH (IU/L)	1.000	0.996–1.004	0.815

VIF (variance inflation factor) values for all variables in the models ranged from 1.2 to 1.9, indicating no significant multicollinearity.

After adjusting for these confounders, average uric acid level remained a significant independent predictor for acute kidney injury (aOR: 3.21, 95% CI: 2.50–4.12, *p* < 0.001) and neurological seizures (aOR: 4.10, 95% CI: 2.95–5.70, *p* < 0.001). Average uric acid also remained predictive for vision loss (aOR: 2.25, 95% CI: 1.62–3.13, *p* < 0.001). Average LDH level, however, did not retain independent predictive significance for most morbidities after adjustment, suggesting it may be a confounder rather than an independent driver for those specific outcomes.

## Discussion

Preeclampsia is one of the most significant causes of mortality and morbidity in obstetrics (10). The morbidities resulting from this condition pose challenges not only for patients but also for the healthcare sector. The decrease in patients' quality of life, the likelihood of developing lifelong comorbidities, as well as the burden on the healthcare system in terms of workforce and financial resources, cannot be ignored. Therefore, research continues to predict and prevent preeclampsia and its outcomes.

A significant consideration for our findings is the cohort's homogeneity; 79% of our preeclampsia group was of Middle Eastern origin. This provides valuable data for this specific demographic but limits generalizability. UA metabolism, baseline levels, and their association with hypertension are known to vary significantly across different ancestries, such as in African or Asian populations (11). Therefore, the specific cutoffs identified in our study (e.g., UA ≥ 6.3 mg/dL for AKI) should not be extrapolated to other ethnic groups without dedicated multicenter validation in those diverse cohorts. The significant difference in BMI between our cohorts is expected, as obesity is a well-documented risk factor for preeclampsia. However, our multivariable analysis confirmed that UA remains a significant predictor of morbidity even after adjusting for BMI, suggesting that its utility is not merely a reflection of maternal weight. Additionally, our finding that the preeclampsia group had significantly lower gravidity and parity is consistent with the well-established association between lower parity (nulliparity) and an increased risk of developing preeclampsia (12).

In the study, the relationship between UA and LDH levels and the mortality and morbidity caused by preeclampsia was investigated. Statistical evaluations showed that UA and LDH levels were significant predictors for some morbidities associated with preeclampsia (acute kidney injury, pulmonary edema, neurological seizures, and vision loss), though not for all complications. No maternal mortality due to preeclampsia was observed during the retrospective period examined in our hospital. Therefore, the relationship between UA and LDH levels and maternal mortality could not be evaluated.

Patients with HELLP syndrome were excluded from the study, as hemolysis-related LDH elevation could confound the association between biomarkers and preeclampsia-specific outcomes.

In a study conducted by Moharana et al. (13) in 2023 with 1,200 patients, it was shown that LDH and UA levels could be associated with the severity of preeclampsia. Moreover, it was suggested that these levels (LDH > 800 IU/L and UA > 6 mg/dL) could also provide insight into the outcomes of the disease, similar to what we observed in our study (13). The fact that their study, conducted with a larger group, yielded results similar to ours supports the reliability of our findings.

In another study conducted by Shivamurthy and Smanjunath (14) in 2020 with 100 patients, it was demonstrated that LDH and UA levels were significant for predicting maternal and fetal outcomes following preeclampsia. Although their study involved a smaller sample group compared to ours, their results aligned with ours, strengthening the reliability of our data. It is important to note that the scope of this study was intentionally focused on severe maternal morbidities, as these represent the most critical and immediate life-threatening outcomes for the mother. While fetal outcomes such as fetal growth restriction, low birth weight, and stillbirth are significant complications of preeclampsia, they were considered beyond the scope of this particular investigation. Our aim was to specifically evaluate the predictive value of UA and LDH for maternal end-organ damage. Research on the correlation of these biomarkers with neonatal outcomes is a valuable direction for future studies.

A study conducted by Sarah and Patil (15) in 2019 with 50 patients demonstrated that LDH and UA biomarkers could be cost-effective and useful for predicting maternal and fetal outcomes in preeclampsia. Their study included 50 preeclamptic patients and 50 healthy pregnant women, with an age range of 18–35 years. LDH and UA levels were compared using a single blood sample. In contrast, our study involved a larger group of 200 patients and 200 controls, with measurements of UA and LDH levels taken throughout hospitalization to obtain more reliable results. Moreover, unlike their study, our research specifically focused on maternal outcomes.

In a 2017 study by Talwar et al. (16), LDH was identified as a prognostic factor, with higher levels correlating with elevated blood pressure in preeclamptic patients. While our findings align in that LDH levels were significantly higher in the preeclampsia group, we did not observe a direct correlation between LDH levels and blood pressure values. This discrepancy could be attributed to several factors, including differences in population genetics, the timing of biomarker measurement relative to blood pressure peaks, or variations in the clinical management of hypertension, which could modulate blood pressure readings independently of systemic endothelial damage reflected by LDH. Our study's focus on *adjusted* multivariable models, whereas the Talwar et al. (16) study reported a simpler correlation, may also account for this difference.

Notably, no significant difference in pre- and post-delivery hemoglobin levels was observed between groups, despite the significantly higher cesarean delivery rate in the preeclampsia group. This may be attributed to the strict exclusion of HELLP syndrome patients, who typically exhibit pronounced hemolysis and are at higher risk for significant postpartum blood loss.

It is crucial to properly contextualize the role of LDH. Our multivariable analysis suggests UA is a more robust independent predictor. However, this does not dismiss the mechanistic importance of LDH, which is a known marker of endothelial damage and hemolysis. The loss of its independent significance after adjusting for confounders like blood pressure suggests that LDH elevation may be secondary to hypertension-induced tissue injury rather than a primary mediator of organ dysfunction itself, as suggested by others (17). Therefore, LDH remains a critical marker of overall disease severity, even if UA provides superior independent risk stratification. Regarding the elevation of LDH, we considered other potential causes of red cell hemolysis. While various disorders can raise LDH, the absence of primary hematological disease in our cohort and the correlation with other preeclampsia-specific markers suggest that the LDH levels observed primarily reflect the systemic endothelial damage and microangiopathic hemolysis inherent to the disease process.

In a 2012 study conducted by Wu et al. (18) with 249 singleton pregnancies, it was shown that UA levels were significant for predicting preeclampsia and its outcomes in patients with gestational hypertension. The study found that higher UA levels significantly increased the risk of developing preeclampsia and that a higher standard deviation in UA levels increased the risk of preeclampsia by 23 times (18). In our study, higher UA levels were similarly associated with maternal morbidity.

In a retrospective study by Vazquez-Alaniz et al. (19) conducted between 2008 and 2017, it was demonstrated that LDH could predict the severity of preeclampsia and be used as a diagnostic marker. In our study, we similarly showed that UA levels, and to a lesser extent LDH levels, could be predictive of maternal outcomes.

We acknowledge that measuring biomarkers early in pregnancy could provide valuable information for predicting the development of preeclampsia. However, our study focused on the relationship between biomarkers and the severity of established preeclampsia. Future research should investigate the role of these biomarkers in the early prediction of preeclampsia, potentially enhancing proactive management strategies. A formal *a priori* power analysis was not conducted, which is a common limitation in retrospective studies. The sample size was pragmatically determined by the number of eligible patient records available at our tertiary care center during the five-year study period (2017–2022). Nevertheless, the cohort of 200 preeclamptic patients and 200 healthy controls is comparable to or larger than that of many similar published studies, and our analysis yielded statistically significant results for several key outcomes, suggesting the sample size was sufficient to detect these associations.

A key addition of our study is the reporting of predictive values and correlation coefficients, which provides deeper clinical context. For instance, the high negative predictive value (NPV) of average uric acid for acute kidney injury (97.8%) and vision loss (98.3%) suggests that these biomarkers are exceptionally useful for ruling out complications. A patient with a normal UA level can be considered at very low risk for these specific outcomes, potentially reducing the need for more intensive monitoring. Conversely, the moderate positive predictive values (PPV), such as 52.3% for kidney injury, highlight that while an elevated biomarker is a significant warning sign, it should not be used as a standalone diagnostic tool, underscoring the need for comprehensive clinical evaluation. Furthermore, the strong correlation coefficients (e.g., ρ = 0.72 for seizures) quantitatively confirm the robust relationship between hyperuricemia and the severity of preeclamptic complications, moving beyond simple association. It is important to note that the extremely high NPVs reported in this study are significantly influenced by the low prevalence of these complications in our cohort. While these values suggest a strong potential for ruling out severe morbidity, they must not be used as standalone diagnostic tools. Clinical decision-making should continue to rely on a comprehensive assessment of all clinical and laboratory parameters. Our findings are consistent with observational data regarding the diagnostic utility of uric acid in preeclampsia. Corominas et al. (22) observed significant elevations in serum uric acid levels in women who developed preeclampsia, particularly those with early-onset disease or intrauterine growth restriction. Notably, their research indicates that uric acid ratios (UAr) offer a high negative predictive value, functioning as a potent parameter for ruling out preeclampsia, which underscores the potential cost-effectiveness of this biomarker in clinical risk stratification.

Our study has several limitations that must be acknowledged. First, its retrospective, single-center design may introduce selection bias. Second, the analysis relied on mean and maximum biomarker values rather than continuous measurements, which might not fully capture the dynamic changes over time. Third, our control group selection method choosing patients who delivered immediately after a preeclamptic patient is a significant limitation and could introduce selection bias. While intended to control for temporal variations in hospital practice, this method is less robust than propensity score matching (PSM). As PSM was not feasible with the available retrospective dataset, this confounding factor must be considered when interpreting the differences between the groups. Finally, due to the retrospective nature of the study, we could not control for unmeasured confounding variables. Specifically, we could not adjust for prophylactic aspirin use or specific antihypertensive regimens, both of which are known to modify preeclampsia severity. While existing literature suggests that UA and LDH levels often remain predictive even after adjustment for aspirin use (20, 21), this remains an important unmeasured confounder in our cohort. Future prospective, multicenter studies are needed to confirm our findings in a more controlled setting. Furthermore, our study is predictive, not causal; it demonstrates a strong correlation, but it cannot definitively conclude whether hyperuricemia is a cause of end-organ damage or merely a marker of it. Additionally, our power analysis was *post-hoc*, which is inherently less robust than an *a-priori* calculation, although it did confirm sufficient power for our primary outcomes. Furthermore, the multivariable model for neurological seizures (aOR: 4.10) must be interpreted with extreme caution. Given the very small number of seizure events (*n* = 4), this finding is exploratory and at high risk of statistical overfitting. In light of these methodological constraints, particularly the retrospective nature and the specific control group selection, our findings represent associations that require validation in more robust, prospective study designs.

Based on our findings, we hypothesize that the routine monitoring of UA and LDH levels in pregnant women diagnosed with preeclampsia can aid in the early identification of patients at higher risk for adverse maternal outcomes. This proactive approach could facilitate timely interventions, such as closer monitoring in an intensive care setting or earlier delivery, potentially leading to improved maternal and fetal outcomes. Further research is needed to validate this hypothesis. A key future direction would be to develop and prospectively validate a clinical risk score that integrates UA, LDH, and other key clinical parameters to provide a more robust and actionable tool for predicting maternal morbidity.

Based on our findings, we propose the following hypothesis-generating clinical risk-stratification approach, which requires prospective validation in future studies: 1) For preeclamptic patients with an average UA level < 6.1 mg/dL, the observed risk of vision loss is exceptionally low (NPV 98.3%), though standard preeclampsia surveillance must continue. 2) For patients with an average UA level < 8.0 mg/dL, the observed risk of eclamptic seizures is similarly low (NPV 100% in this cohort), suggesting UA may be a tool to help rule out these specific outcomes. 3) A patient with an average UA level ≥8.0 mg/dL (100% sensitivity for seizures) should be considered at very high risk, warranting urgent neurologic evaluation and consideration for seizure prophylaxis.

## Conclusion

In this study, while no preeclampsia-related maternal mortality was observed, serum uric acid and LDH levels were significantly elevated in preeclamptic patients. Uric acid, in particular, demonstrated strong predictive utility for severe maternal morbidities, showing high negative predictive values for ruling out acute kidney injury, neurological seizures, and vision loss. We conclude that monitoring UA and LDH is an inexpensive and accessible strategy that can aid clinical risk stratification in preeclamptic patients. Further large-scale prospective studies are needed to validate these findings and integrate them into clinical management algorithms. However, given the exploratory nature of some analyses and the inherent risks of selection bias, these biomarkers should be viewed as adjuncts to, rather than replacements for, established clinical monitoring protocols.

## Data Availability

The raw data supporting the conclusions of this article will be made available by the authors, without undue reservation.
